# Posttraumatic Stress Disorder, Suicidal Ideation, and Suicidal Self-Directed Violence Among U.S. Military Personnel and Veterans: A Systematic Review of the Literature From 2010 to 2018

**DOI:** 10.3389/fpsyg.2020.01998

**Published:** 2020-08-26

**Authors:** Ryan Holliday, Lauren M. Borges, Kelly A. Stearns-Yoder, Adam S. Hoffberg, Lisa A. Brenner, Lindsey L. Monteith

**Affiliations:** ^1^Rocky Mountain Mental Illness Research, Education and Clinical Center for Veteran Suicide Prevention, Aurora, CO, United States; ^2^Department of Psychiatry, University of Colorado Anschutz Medical Campus, Aurora, CO, United States; ^3^Department of Physical Medicine and Rehabilitation, University of Colorado Anschutz Medical Campus, Aurora, CO, United States; ^4^Department of Neurology, University of Colorado Anschutz Medical Campus, Aurora, CO, United States

**Keywords:** posttraumatic stress disorder, suicidal ideation, suicide attempt, suicide, military personnel, veteran, systematic review

## Abstract

Rates of suicide and posttraumatic stress disorder remain high among United States military personnel and veterans. Building upon prior work, we conducted a systematic review of research published from 2010 to 2018 regarding: (1) the prevalence of suicidal ideation, suicide attempt, and suicide among United States military personnel and veterans diagnosed with posttraumatic stress disorder; (2) whether posttraumatic stress disorder was associated with suicidal ideation, suicide attempt, and suicide among United States military personnel and veterans. 2,106 titles and abstracts were screened, with 48 articles included. Overall risk of bias was generally high for studies on suicidal ideation or suicide attempt and low for studies on suicide. Across studies, rates of suicidal ideation, suicide attempt, and suicide widely varied based on study methodology and assessment approaches. Findings regarding the association between posttraumatic stress disorder diagnosis with suicidal ideation and suicide were generally mixed, and some studies reported that posttraumatic stress disorder was associated with lower risk for suicide. In contrast, most studies reported significant associations between posttraumatic stress disorder and suicide attempt. These findings suggest complex associations between posttraumatic stress disorder and suicidal ideation, suicide attempt, and suicide, which are likely influenced by other factors (e.g., psychiatric comorbidity). In addition, most samples were comprised of veterans, rather than military personnel. Further research is warranted to elucidate associations between posttraumatic stress disorder and suicidal ideation, suicide attempt, and suicide, including identification of moderators and mediators of this relationship. Addressing this among United States military personnel, by gender, and in relation to different trauma types is also necessary.

## Introduction

Within the United States (U.S.), suicide remains a significant public health concern, with the Centers for Disease Control and Prevention (CDC) recently reporting it as the tenth overall leading cause of death (Heron, 2018). Risk for suicide is especially pronounced among U.S. military personnel and veterans, among whom adjusted suicide rates have, at times, outpaced suicide rates in the general U.S. non-veteran adult population (Reimann and Mazuchowski, 2018; Department of Veterans Affairs, 2019). As such, preventing suicide among military personnel and veterans remains a top clinical priority of the Departments of Defense and VA.

Efforts have been made to understand why U.S. military personnel and veterans are at increased risk for suicide, compared to civilians. Although suicide is understood to be etiologically complex, one potential conduit of increased risk is through traumatic experiences and their sequelae. Military personnel and veterans experience trauma with heightened propensity, both in terms of military- and non-military-related trauma, such as combat-related experiences, military sexual assault, childhood abuse, and intimate partner violence (Gates et al., 2012; Lehavot et al., 2018). Military personnel and veterans also experience high rates of posttraumatic stress disorder (PTSD) (Gates et al., 2012; Lehavot et al., 2018). As such, researchers have posited elevated rates of PTSD as a potential explanation for suicidal ideation (SI), suicide attempt (SA), and suicide among service members and veterans (Pompili et al., 2013). Indeed, prior systematic reviews and meta-analyses of the relationship between PTSD and suicide have reported significant associations between PTSD and suicide risk (Krysinska and Lester, 2010; Kanwar et al., 2013; Panagioti et al., 2015). However, to date, only one such review has focused specifically on military personnel and veterans (Pompili et al., 2013).

Pompili et al. (2013) conducted a systematic review of literature published from 1980 to 2010 regarding the association between PTSD with SI and suicidal self-directed violence (S-SDV; e.g., suicide attempt, suicide) among U.S. and Canadian military personnel and veterans. Based on their review of 18 studies, they concluded that PTSD was associated with SI, SA, and suicide. Pompili et al. further noted, however, that PTSD was associated with several mental health outcomes (e.g., psychiatric comorbidity), which may have partially accounted for the reported associations. As such, it remains difficult to ascertain to what extent PTSD independently explains heightened risk for SI, SA, and suicide among U.S. military personnel and veterans.

While the aforementioned systematic review was seminal in its focus on military personnel and veterans, the review focused on “war-related PTSD” (e.g., combat-related), limiting inference regarding PTSD from other prevalent military- or non-military related traumatic exposures (e.g., military sexual assault, childhood abuse). Another limitation of the prior review was the inclusion of studies focused on PTSD symptoms, limiting the ability to draw precise inferences regarding PTSD diagnosis, as those without a diagnosis of PTSD may have been included. In addition, since 2010, veterans from the recent conflicts in Afghanistan and Iraq have experienced high rates of deployment-related experiences, such as combat and sexual assault (Street et al., 2009; Vasterling et al., 2010; Barth et al., 2016). While some of these experiences are not necessarily unique to deployment (e.g., sexual assault can occur during training or while stateside), deployment can disrupt pre-deployment life and family functioning. For example, Paley et al. (2013) noted that deployment can introduce a number of challenges, including disruptions in family routine, extended separation from friends and family, and parenting challenges following return due to mental health sequelae. These disruptions appear particularly salient in driving PTSD symptomatology following military-related trauma (Polusny et al., 2014). This, in turn, may result in differing clinical presentations in more recent research with military personnel and veterans that could impact associations of PTSD with SI and S-SDV. As such, an updated review of the literature published since 2010 is critical.

Finally, a number of prior reviews discussed SI, SA, and suicide as categorically-similar constructs, rather than differentiating between these outcomes. This is problematic to discerning optimal clinical care to mitigate risk among service members and veterans with PTSD (Holliday et al., 2019). The VA and Department of Defense have mandated a specific classification system and nomenclature for suicide, the Centers for Disease Control and Prevention's (CDC) Suicidal Self-Directed Violence Classification System (Crosby et al., 2011), to distinguish these constructs. The CDC defines suicidal ideation as “thoughts of engaging in suicide-related behavior” (p. 90), suicide attempt as “a non-fatal self-directed potentially injurious behavior with any intent to die as a result of the behavior…which may or may not result in injury” (p. 21), and suicide as “death caused by self-directed injurious behavior with any intent to die as a result of the behavior” (p. 23). As these constructs have distinct underlying theoretical underpinnings, as well as divergent risk factors (Joiner, 2007; Nock et al., 2008; Klonsky and May, 2015; Klonsky et al., 2016; May and Klonsky, 2016), delineating the extent to which a diagnosis of PTSD is associated with SI, SA, and suicide remains crucial.

The current systematic review aimed to address these limitations and provide an enhanced update of research examining the association between PTSD diagnosis and SI, SA, and suicide among U.S. military personnel and veterans. To update prior work (c.f. Pompili et al., 2013), this review focused on literature published between 2010 and 2018. Specifically, we focused on two key questions (KQ):

KQ1. What is the prevalence of SI, SA, and suicide among U.S. military personnel and veterans with a diagnosis of PTSD?
KQ2. Is PTSD diagnosis associated with SI, SA, and suicide among U.S. military personnel and veterans?

## Methods

Methods and presentation of results map onto the Preferred Reporting Items for Systematic Reviews and Meta-Analyses (PRISMA) guidelines (Moher et al., 2009). This systematic review was registered in PROSPERO (CRD42018089267). We conducted a systematic search of literature published between January 1, 2010 and April 25, 2018 in the following electronic databases: OVID MEDLINE, EMBASE, OVID PsycINFO, Web of Science, Cumulative Index to Nursing and Allied Health Literature (CINAHL), Published International Literature On Traumatic Stress (PILOTS), and Cochrane Library. The search strategy was developed using medical subject heading terms (MeSH) and relevant text and key words in OVID MEDLINE, then replicated in each database (see Supplementary Table 1 for additional information). Google Scholar was also searched to identify gray literature studies that met inclusion criteria but had been published outside traditional academic distribution channels or were not yet indexed in electronic databases. Reference lists were also mined for relevant publications not already identified.

Eligibility criteria were defined in accordance with the Population, Intervention, Comparators, Outcomes, Timing/Setting (PI[E]COTS) framework (Moher et al., 2009; Matchar, 2012). In particular, inclusion criteria were as follows: Population(s): U.S. military personnel and/or veterans; Intervention/Exposure(s): (1) Assessment and diagnosis of PTSD and (2) assessment or documentation of SI, SA, or suicide; Comparator(s): A comparison group was not required for KQ1; however, for KQ2, a comparator of no PTSD diagnosis was required; Outcome(s): Prevalence of SI, SA, or suicide among those with PTSD (KQ1); reported on the association between PTSD and SI, SA, or suicide (KQ2); and Timing/Setting: No restrictions based on timing, setting, or study design. Additional criteria for inclusion were: (1) presentation of original study data in a peer-reviewed journal article; (2) adequate data to address KQ1 and/or KQ2 (i.e., reported rate and/or association between PTSD and SI, SA, or suicide within text); (3) the full-text article was in English; and (4) published between January 1, 2010 and April 25, 2018. Exclusion criteria were as follows: (1) duplicate datasets (i.e., re-analysis of a previously reported dataset) and (2) inadequate data (i.e., inability to calculate rates or associations based on content reported within the manuscript); (3) dissertations, conference proceedings, commentaries, editorials, letters, books, book chapters, duplicate datasets, and reviews.

Databases were searched sequentially on the same day. Complete citations were exported and de-duplicated using EndNote X8 reference management software (Thomson Reuters, New York City, NY, USA). References were then exported into Covidence review software. Review of studies for inclusion was based on previously-used systematic review frameworks, including those used by the study team (Hoffberg et al., 2020).

For the PRISMA screening stage, at least two reviewers (RH, LMB, KSY, LAB) independently screened each title and abstract for retrieval. When not in agreement, a third reviewer (LLM) evaluated the record for the final retrieval decision. All co-authors have experience conducting research on PTSD and SI and S-SDV among U.S. veterans and have previously published in this domain (e.g., Brenner et al., 2011a; Holliday et al., 2018b; Barnes et al., 2019; Monteith et al., 2019).

Covidence was also used to evaluate records selected for the PRISMA eligibility stage of the review. Each full-text record was assessed by at least two reviewers (RH, LMB, KSY, LAB). The decision process was stepwise and based on the PICOTS model. Each reviewer progressed through the PICOTS decision tree until either all inclusion criteria were met or the record was excluded for a particular reason. Any disagreements at this stage were similarly resolved by a third blind reviewer (LLM).

Data from full-text articles selected for inclusion were abstracted into tables by three authors (RH, LMB, and LLM). Conflicts were resolved by group consensus, with RH making the final determination for inclusion. The preliminary data abstraction template was tested before being finalized. The following information was extracted: source article, population/sample (e.g., composed of military personnel or veterans, proportion of males and females within the sample, index trauma), measurement of PTSD, measurement of SI/SA/suicide, prevalence of SI/SA/suicide among those diagnosed with PTSD (KQ1), association of PTSD to SI/SA/suicide (KQ2), and additional relevant results. As variability of study designs and outcome measurements precluded a meta-analytic approach, a descriptive synthesis approach was conducted (McKenzie and Brennan, 2019). For each study included, the sample was described based on composition (i.e., military personnel or veteran) and proportion of males/females; however, due to infrequent reporting across studies, index trauma could not consistently be assessed and was thus not reported.

Included articles were classified by study design using the Taxonomy of Study Design Tool (Hartling et al., 2010) in a custom Research Electronic Data Capture (REDCap) database (Harris et al., 2009). Study design was assessed independently (KSY, ASH), with conflicts resolved by a third author (RH). Each of these authors had prior experience conducting systematic reviews or meta-analyses (Bahraini et al., 2013; Hoffberg et al., 2018, 2020; Holliday et al., 2018a; Creech et al., 2019).

After reaching consensus on study design, risk of bias was assessed using the Effective Public Health Practice Project (EPHPP) quality assessment tool for quantitative studies (Thomas et al., 2004). Bias items included selection bias, study design, confounders, blinding, data collection, withdrawals/dropouts, and other sources (e.g., no disclosure of conflicts of interest). Each of these domains, if applicable, were rated as having a low, moderate, or high risk of bias based on standard guidelines (see Thomas et al., 2004 for additional information). An overall risk of bias rating was then generated using standard guidelines (Effective Public Health Practice Project, 1998a,b), such that studies rated as having no high risk of bias domain ratings were classified as low overall risk of bias, studies having up to one high risk of bias domain rating were classified as moderate overall risk of bias, and studies having two or more high risk of bias domain ratings were classified as high overall risk of bias. Risk of bias was evaluated independently by KSY and ASH, with discrepancies discussed among KSY, ASH, and RH until achieving consensus. Both KSY and ASH had prior experience evaluating risk of bias in prior systematic reviews (e.g., Hoffberg et al., 2018, 2020).

## Results

### Study Selection

**Figure 1:**
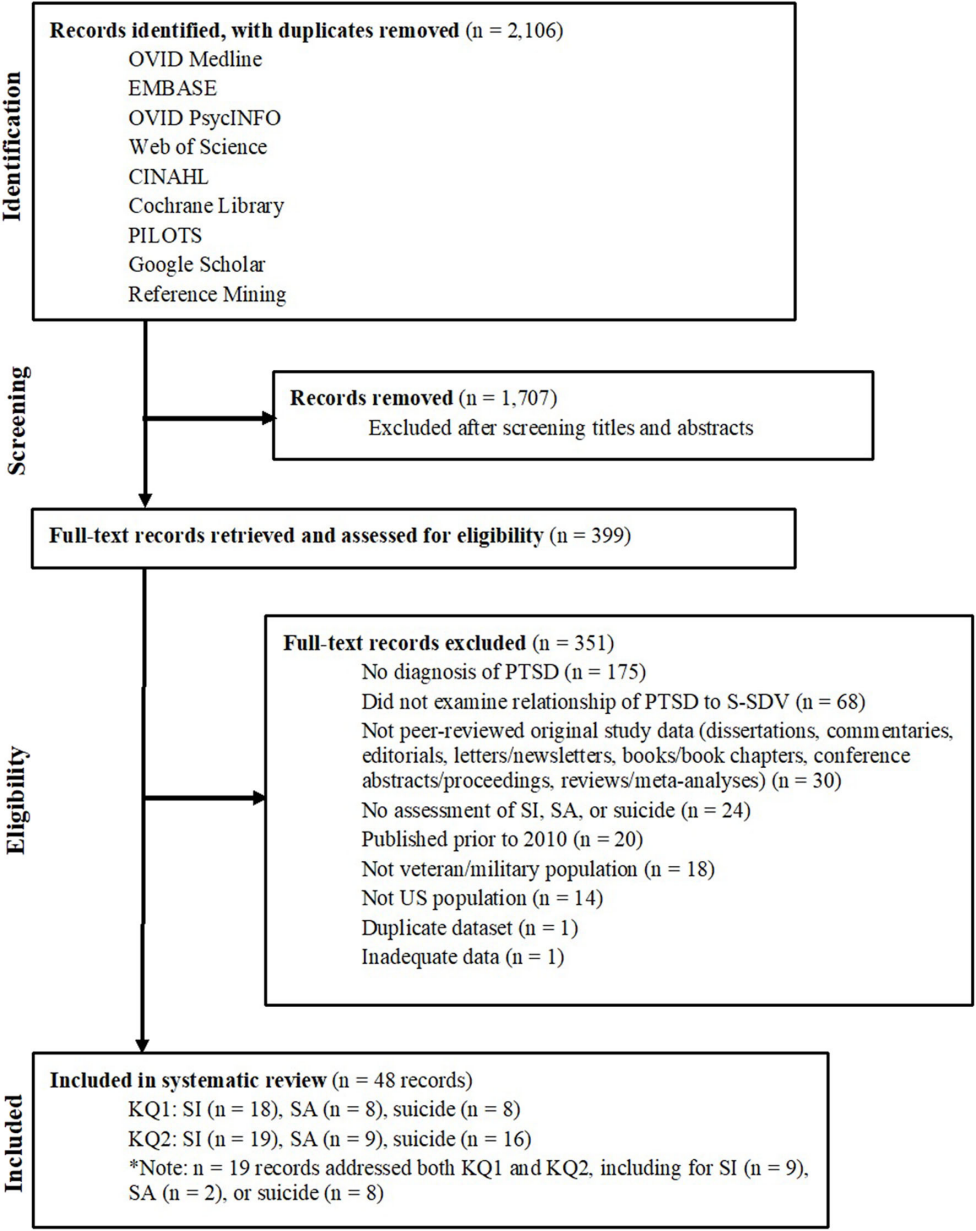
PRISMA literature flow diagram.

As depicted in Figure 1, 2,106 titles and abstracts identified in electronic searches were screened after removing duplicates. Following screening, 399 full-text articles were assessed for eligibility. Based on our PICOTS criteria, 351 of these full-text articles were subsequently excluded, resulting in 48 articles included for KQ1 and/or KQ2. Risk of bias for these articles is reported in Table 1.

**Table 1:**
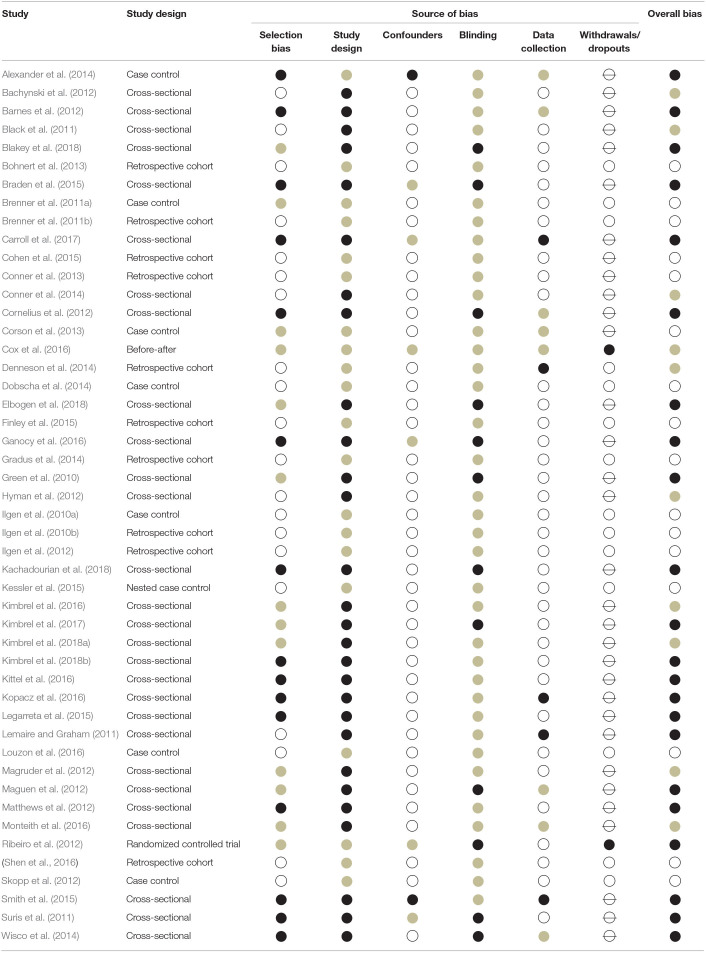
Risk of bias ratings for each study (*N* = 48).

*Risk of bias: ○ = low; •= moderate; •= high; ∘ = not applicable*.

### Synthesis of Literature

#### KQ1: Prevalence of SI Among Military Personnel and Veterans With PTSD

Eighteen studies reported on the prevalence of SI among U.S. military personnel or veterans diagnosed with PTSD. Rates of SI across studies ranged from 1.98 to 71.89% (Table 2). However, the overwhelming majority of included studies (*n* = 13) had a high overall risk of bias. Three had an overall moderate risk of bias and reported rates of SI ranging from 35.71 to 44.90% (Magruder et al., 2012; Denneson et al., 2014; Cox et al., 2016). Only two studies had a low overall risk of bias, reporting that 3.21–34.81% of veterans with PTSD had SI (Corson et al., 2013; Cohen et al., 2015). Of studies relying on interview or assessment measures, current/recent rates of SI ranged from 1.98 to 44.9%, whereas lifetime rates varied more broadly, from 17.39 to 71.89%. All of these studies focused on veterans; none focused exclusively on military personnel. Studies evaluating lifetime SI often comprised fairly small clinical samples, whereas studies reporting on more recent SI involved much larger samples, typically comprising treatment-seeking Operation Enduring Freedom/Operation Iraqi Freedom (OEF/OIF) veterans.

**Table 2:** SI among military personnel and veterans with PTSD (*n* = 28).

**Study**	**Population/sample**	**Measurement of PTSD**	**Measurement of SI**	**Prevalence of SI among those with PTSD**	**Relationship of PTSD to SI**	**Notes**
Barnes et al. (2012)	92 male OEF/OIF veterans with combat-related PTSD presenting for VA outpatient PTSD treatment.	Clinical interview (including CAPS for DSM-IV)	Interview question: “Have you had thoughts about death or about killing yourself?” (Yes/No; lifetime)	16 (17.39%) endorsed lifetime SI.^*^	Did not analyze relationship.	Exclusion criteria of TBI-related LOC > 30 min or PTA > 24 h.
Blakey et al. (2018)	667 OEF/OIF/OND veterans (81.0% male), active duty personnel, and National Guard and Reserve members who had served since September 11, 2001 and endorsed chronic pain.	SCID-IV	BSS and/or BDI-II item 9	Not reported.	PTSD was associated with SI on a bivariate level, *P* = 0.17, *p* <0.01. After accounting for demographics, chronic pain intensity and interference, and comorbid MH conditions, PTSD remained a significant correlate (OR = 2.25, 95% CI: 1.18–4.24, *p* = 0.01).	Disagreement in SI scoring on BSS and BDI-II item 9 was present for 65 (51.18%) of SI data.^*^
Braden et al. (2015)	110 veterans (90.9% male) with a primary mood disorder diagnosis.	SCID-IV	BSS	Not reported.	Accounting for demographics, PTSD was not associated with SI, *p* > 0.05; however, poorer physical health, prior psychiatric hospitalization, and past SA were associated in this sample.	Participants were excluded for a diagnosis of bipolar disorder, schizophrenia, dementia, organic brain damage, and intellectual disability.
Carroll et al. (2017)	217 male Iraq/Afghanistan-era veterans with combat-related PTSD entering a PTSD residential program.	“Intake procedures” (measure not reported)	Interview question: “Have you ever had serious thoughts of committing suicide?” (Yes/No; lifetime)	156 (71.89%) endorsed lifetime SI.	Did not analyze relationship.	Inclusion was based on presence of combat-related PTSD diagnosis. Participants were excluded based on presence of psychotic symptoms, unwillingness to stop using drugs/alcohol, and medical conditions impeding engagement in treatment.
Cohen et al. (2015)	186,460 OEF/OIF/OND veterans (90.6% male) with PTSD and an initial VHA visit from January 1, 2007 to September 30, 2011.	VA medical record	VA medical record	5,988 (3.21%) with PTSD had documented SI.^*^	Did not analyze relationship.	Veterans with a diagnosis of bipolar disorder or schizophrenia were excluded.
Cornelius et al. (2012)	101 veterans (89.1%) using VHA outpatient behavioral health services.	SCID-IV	BDI item 9	2 (1.98%) of those with PTSD endorsed SI.	PTSD was not associated with SI at a bivariate level, χ^2^= 0.08, *p* = 0.773.	
Corson et al. (2013)	1,340 veterans (89.4% male) using VHA care with a positive depression screen (PHQ-2).	VA medical record	PHQ-9 item 9 and VA Pocket Card Risk Assessment	235 (34.81%) of those with PTSD endorsed SI.^*^	Bivariate associations between PTSD and SI were not significant, *p* = 0.06. After controlling for age, sex, marital status, branch, PHQ-2 scores, SUD, and when SI was assessed, PTSD was not predictive of SI (AOR = 1.18, 95% CI: 0.93–1.50, *p* = 0.186). Rather, the only significant MH diagnoses predictive of SI were depression and bipolar/schizophrenia.	
Cox et al. (2016)	289 veterans (89.0% male) receiving Prolonged Exposure Therapy from a VHA clinic.	CAPS for DSM-IV or PSS-I	BDI-II item 9	127 (43.94%) endorsed SI.	Did not analyze relationship.	Inclusion criterion of at least 2 timepoints of SI measurement.
Denneson et al. (2014)	465 OEF/OIF veterans (87.5% male) new to VA care who screened positive for depression.	VA medical record	PHQ-9 item 9 and/or VA Pocket Card Risk Assessment	80 (35.71%) with documented PTSD screened positive for SI.^*^	PTSD diagnosis was not associated with SI on a bivariate, *p* = 0.07, or multivariate level (AOR = 1.10; 95% CI: 0.72-1.69, *p* = 0.67). Multivariate results found depression to be a significant correlate adjusting for sex, age, race/ethnicity, rurality, marital status, SI assessment by MH clinician, and MH comorbidity.	
Elbogen et al. (2018)	2,543 Iraq/Afghanistan-era veterans, active duty personnel, and reserve forces (80.2% male).	SCID-IV	BSS	205 (28.55%) with PTSD endorsed SI.^*^	Accounting for demographics and resiliency, childhood abuse, pain, and depression, but not PTSD (OR = 1.34, 95% CI: 0.94–1.90, *p* = 0.106), were associated with SI.	Most participants were registered at a VA medical facility.
Finley et al. (2015)	211,652 veterans (86.4% male) receiving VA care during FY 2009-2011.	VA medical record	VA medical record	Not reported.	Accounting for demographics and MH comorbidity, PTSD was associated with SI (OR = 2.3, 95% CI: 2.0–2.6, *p* <0.01), with risk increasing in the presence of MH comorbidity (e.g., depression, SUD).	
Ganocy et al. (2016)	418 Army National Guard personnel (88.0% male) participating in a longitudinal cohort study.	CAPS for DSM-IV (lifetime)	C-SSRS	Not reported.	Bivariate associations between lifetime PTSD and SI were significant, *r* = 0.24, *p* <0.01.	The number of participants endorsing lifetime PTSD (28; 6.8%), as well as SI (32; 7.7%), were small.
Green et al. (2010)	497 deployed OEF/OIF veterans (83.1% male).	SCID-IV	BSS	41 (21.69%) with PTSD had past-week SI.	Those diagnosed with PTSD reported higher BSS scores at a bivariate level, *t* = −4.98, *p* <0.001.	Veterans without lifetime trauma exposure were excluded.
Kachadourian et al. (2018)	93 veterans (93.5% male) with comorbid PTSD and alcohol dependence participating in an RCT.	SCID-IV	C-SSRS (lifetime)	60 (63.83%) with PTSD reported lifetime SI.	Did not analyze relationship.	Exclusion criteria of pregnancy, schizophrenia, schizophrenia-type disorders, bipolar disorder, active SI or homicidal ideation, use of medication likely to influence alcohol consumption, and Prazosin contraindication.
Kimbrel et al. (2017)	3,233 Iraq/Afghanistan-era veterans (79.7% male).	SCID-IV (lifetime)	BSS	Not reported.	Adjusting for sex, lifetime depression, lifetime alcohol use disorder, lifetime non-cannabis drug use disorder, lifetime cannabis use disorder, childhood sexual abuse, and combat exposure, lifetime PTSD diagnosis (OR = 2.08, 95% CI: 1.50–2.89, *p* <0.001) as well as depression, childhood sexual abuse, combat exposure, and cannabis use disorder were associated with SI.	The majority of veterans were enrolled in VHA care.
Kimbrel et al. (2018a)	1,143 veterans (95.9% male) seeking PTSD treatment.	SCID-IV	BDI-II Item 9	Not reported.	PTSD was associated with presence of SI (OR = 2.13; 95% CI: 1.43–3.18, *p* <0.05), accounting for gender, age, combat exposure, and NSSI.	
Kittel et al. (2016)	130 (85.3% male) Iraq and Afghanistan-era veterans enrolled in VHA care.	CAPS for DSM-IV	BSS	Not reported.	A bivariate, positive relationship between current PTSD diagnosis and past week SI severity was significant, *p* = 0.002.	Participants were excluded if endorsed SI, intent or plan warranting crisis intervention; none excluded based on this criterion.
Kopacz et al. (2016)	472 (94.1% male) veterans admitted for treatment at one of two VA PTSD Residential Rehabilitation Programs	Clinical interview and unreported assessment measures	Affirmative response to: “Have you ever had serious thoughts of committing suicide?” (lifetime)	336 (71.19%) reported a lifetime history of SI^*^	Not reported.	
Legarreta et al. (2015)	95 veterans (77.9% male) who reported at least one lifetime *DSM−5* Criterion A traumatic event.	Checklist created using SCID-IV, Trauma Symptom Index, HAM-D, Profile of Mood States, and methodical query	C-SSRS (lifetime)	47 (49.47%) with PTSD endorsed lifetime SI.^*^	PTSD was associated with lifetime SI at a bivariate level, χ^2^ = 5.05, *p* = 0.03.	Exclusion criteria of major sensorimotor handicaps, IQ <80, and psychosis. Unvalidated measure of PTSD.
Lemaire and Graham (2011)	1,740 OEF/OIF veterans (84.1% male) participating in routine VA mental health screening.	VA medical record	Clinical interview	64 (12.88%) with PTSD reported SI.^*^	PTSD was associated with documented SI at the bivariate level (OR = 10.02, 95% CI: 4.02–24.97, *p* <0.001). PTSD was not significant and thus not included in the final forward stepwise multivariate regression predicting SI. Rather, depressive disorders, social support, gender, and prior SA were noted correlates in this model.	
Magruder et al. (2012)	816 veterans (83.9% male) randomly selected from 4 Southeast VA hospitals.	CAPS for DSM-IV	MINI Suicidality module	44 (44.90%) with PTSD reported current SI.^*^	PTSD was positively associated with current SI at the bivariate level, *p* <0.01. In multivariate analyses, PTSD was associated with SI in the presence of other comorbidities (depression, anxiety disorders; OR = 4.02, 95% CI: 1.95–8.29, *p* <0.05).	Women oversampled.
Maguen et al. (2012)	259 male Vietnam veterans participating in a national survey.	SCID for DSM-III-R	Affirmative response: “Have you ever felt so low that you thought of committing suicide?” (lifetime)	Not reported.	After adjusting for demographic variables, killing experiences, depression, SUD, and PTSD (OR = 3.42, 95% CI = 1.09–10.73, *p* <0.05) were associated with SI.	Weighted results to population of 1.3 million veterans.
Matthews et al. (2012)	26 male combat-exposed OEF/OIF veterans evaluated at an outpatient mood or psychiatric emergency clinic.	Semi-structured clinical interview	Comprehensive Suicide Risk Assessment (lifetime)	13 (54.16%) with PTSD endorsed lifetime SI.	Did not analyze relationship.	Exclusion criteria of alcohol/substance dependence in past 30 days; lifetime ADHD; psychotic, bipolar, chronic pain disorders; active medical problems; or claustrophobia.
Monteith et al. (2016)	354 (87.6% male) veterans accessing VHA care.	VA medical record	BSS	Not reported.	PTSD was associated with SI presence, *r* = 0.12, *p* <0.05, and severity, *r* = 0.23, *p* <0.01. When age, gender, combat exposure, depressive disorders, PTSD diagnosis, negative affect, past SA, military sexual trauma, and an interactive term of military sexual trauma x gender were included in a regression model, lifetime PTSD diagnosis was no longer associated with SI severity, *B* = 0.08, 95% CI: 0.06–2.06, *p* = 0.80.	
Ribeiro et al. (2012)	311 military personnel (82.0% male) referred for suicide-focused treatment.	DIS	MSSI	Not reported.	PTSD was associated with SI at the bivariate level, *r* = 0.20, *p* <0.01. After accounting for baseline hopelessness, depression, anxiety, drug abuse, alcohol abuse, and insomnia symptoms, PTSD was not associated with SI, *p* > 0.05.	Results did not account for sociodemographic variables (e.g., gender).
Smith et al. (2015)	832 veterans (gender breakdown not reported) entering a VHA or non-profit PTSD residential program.	Prior primary diagnosis of PTSD (measure not reported)	Interview question: “Have you ever had serious thoughts of committing suicide?” (Yes/No; lifetime)	194 (71.06%) reported a lifetime history of SI.^*^	Did not analyze relationship.	Article reported a total sample of 832; however, suicide-related data were only reported for 273 veterans. Exclusion criteria of active psychosis, unwillingness to discontinue substance misuse, and medical conditions that would hinder/prevent engagement in treatment.
Suris et al. (2011)	128 veterans (11% male) currently diagnosed with military sexual assault-related PTSD participating in a PTSD RCT.	CAPS for DSM-IV	BDI-II Item 9	59 (46.09%) of sample endorsed SI.^*^	Did not analyze relationship.	Exclusion criterion of “active suicidality.”
Wisco et al. (2014)	1,649 deployed OEF/OIF/OND veterans (49.9% male).	SCID for DSM-IV (lifetime)	MINI Suicidality module	375 (30.00%) of veterans with a lifetime diagnosis of PTSD reported current SI.^*^	Those with PTSD reported SI more frequently at a bivariate level, V = 0.19, *p* <0.001. After accounting for age, gender, ethnicity, race, combat and postbattle experiences, postdeployment support, depressive symptoms, alcohol problems, and TBI, lifetime PTSD was associated with SI: RR = 2.16, 95% CI: 1.29-3.61, *p* <0.05.	Participants deemed to be at “high suicide risk” were excluded. Oversampled veterans with probable PTSD. Results were significant for both males and females for gender-stratified.

* Calculated using data reported in manuscript.

BDI-II, Beck Depression Inventory-II; BSS, Beck Scale for Suicide Ideation; C-SSRS, Columbia-Suicide Severity Rating Scale; CAPS, Clinician Administered PTSD Scale; DIS, Diagnostic Interview Schedule; DSM-IV, Diagnostic and Statistical Manual of Mental Disorders-IV; FY, fiscal year; HAM-D, Hamilton Rating Scale for Depression; IQ, intelligence quotient; LOC, loss of consciousness; MH, mental health; MINI, Mini International.

*Neuropsychiatric Interview; MSSI, Modified Scale for Suicidal Ideation; NSSI, non-suicidal self-injury; OR, odds ratio; PHQ-2: Patient Health Questionnaire-2; PHQ-9: Patient Health Questionnaire-9; PTA, posttraumatic amnesia; PSS-I, Posttraumatic Symptom Interview; PTSD, posttraumatic stress disorder; RCT, randomized clinical trial; RR, relative risk; SCID for DSM-III-R, Structured Clinical Interview for DSM-III-R; SCID-IV, Structured Clinical Interview for DSM-IV; SUD, substance use disorder; TBI, traumatic brain injury; VA, Department of Veterans Affairs; VHA, Veterans Health Administration*.

#### KQ1: Prevalence of SA Among Military Personnel and Veterans With PTSD

The eight studies on rates of SA among those diagnosed with PTSD reported rates ranging from 0.31 to 38.71% (Table 3). Only one included study was determined to have a low overall risk of bias (Gradus et al., 2014), and one had a moderate overall risk of bias (Kimbrel et al., 2016). Of note, all of the studies on the prevalence of SA among those with PTSD focused on veterans; none reported on rates of SA among military personnel with PTSD.

**Table 3:** SA among Military Personnel and Veterans with PTSD (*n* = 15).

**Study**	**Population/sample**	**Measurement of PTSD**	**Measurement of SA**	**Prevalence of SA among those with PTSD**	**Relationship of PTSD to SA**	**Notes**
Barnes et al. (2012)	92 male OEF/OIF veterans with combat-related PTSD presenting for VA outpatient PTSD treatment.	Clinical interview (including CAPS for DSM-IV)	Interview question: “Have you ever attempted suicide?” (Yes/No; lifetime)	5 (5.43%) reported a history of SA.^*^	Did not analyze relationship	Excluded if loss of consciousness exceeded 30 min or posttraumatic amnesia exceeded 24 h.
Brenner et al. (2011a)	241 veterans (83.0% male) using VHA care.	VA medical record	VA medical record	Not reported.	Bivariate associations between PTSD and SA were significant, χ^2^ = 10.7, *p* = 0.001. In a multivariate regression of PTSD and TBI, PTSD was a predictor of SA (OR = 2.85, 95% CI: 1.55–5.22, *p* = 0.0007).	Veterans with SA history were matched to controls with no SA history, based on gender and age.
Carroll et al. (2017)	217 male Iraq/Afghanistan-era veterans with combat-related PTSD entering a PTSD residential program.	“Intake procedures” (measure not reported)	Interview question: “Have you attempted suicide in your lifetime?” (Yes/No; lifetime)	84 (38.71%) endorsed lifetime SA.	Did not analyze relationship.	Inclusion was based on presence of combat-related PTSD diagnosis. Participants were excluded based on presence of psychotic symptoms, unwillingness to stop using drugs/alcohol, and medical conditions impeding engagement in treatment.
Finley et al. (2015)	211,652 veterans (86.4% male) receiving VA care during FY 2009-2011.	VA medical record	VA medical record	Not reported.	Accounting for demographics and MH comorbidity, PTSD was associated with SA (OR = 1.8, 95% CI: 1.2–2.8, *p* <0.01), with risk increasing in the presence of MH comorbidity (e.g., depression, SUD).	
Gradus et al. (2014)	68,506 veterans (92.1% male) using VHA care.	VA medical record	VA medical record	49 (0.31%) with PTSD had a documented prior SA^*^	Adjusting for marital status, depression, alcohol/drug abuse/dependence, anxiety disorder diagnoses, and intentional self-harm, PTSD was associated with non-fatal intentional self-harm in both males (RR = 3.2, 95% CI = 2.3, 4.5) and females (RR = 16, 95% CI = 4.8, 56).	SA classified as “non-fatal intentional self-harm.”
Kachadourian et al. (2018)	93 veterans (93.5% male) with comorbid PTSD and alcohol dependence participating in an RCT.	SCID-IV	C-SSRS (lifetime)	19 (21.59%) with PTSD reported lifetime SA.	Did not analyze relationship.	Exclusion criteria of pregnancy, schizophrenia, schizophrenia-type disorders, bipolar disorder, active SI or homicidal ideation, use of medication likely to influence alcohol consumption, and Prazosin contraindication.
Kimbrel et al. (2016)	292 veterans (67.5% male) deployed in support of Iraq and Afghanistan conflicts.	CAPS for DSM-IV (lifetime)	C-SSRS (lifetime)	19 (13.1%) with lifetime PTSD endorsed lifetime SA.^*^	Bivariate associations between PTSD and SA not significant, χ^2^ = 2.379, *p* = 0.12. PTSD was not significant in multivariate model that included gender, age, race, sexual orientation, psychiatric diagnoses, combat exposure, and non-suicidal self-injury: OR = 0.364, 95% CI: 0.11–1.22, *p* > 0.05.	Small sample reporting lifetime SA (30; 10.0%)
Kimbrel et al. (2017)	3233 Iraq/Afghanistan-era veterans (79.7% male).	SCID-IV (lifetime)	BSS (lifetime)	Not reported.	Adjusting for sex, lifetime depression, lifetime alcohol use disorder, lifetime non-cannabis drug use disorder, lifetime cannabis use disorder, childhood sexual abuse, and combat exposure, PTSD was associated with SA (OR = 2.60, 95% CI: 1.87–3.61, *p* <0.001).	Most participants were enrolled in VHA care.
Kimbrel et al. (2018b)	292 Iraq/Afghanistan-era veterans (67.5% male).	CAPS for DSM-IV (lifetime)	C-SSRS (lifetime)	Not reported.	After accounting for sociodemographics, combat exposure, traumatic life events, TBI, depression, alcohol use disorder, and non-cannabis drug use disorder, and cannabis use disorder, PTSD was not associated with SA (OR =0.52, 95% CI: 0.18–1.50, *p* > 0.05). Rather, sex, combat exposure, depression, and cannabis use disorder were positively associated with SA.	
Kopacz et al. (2016)	472 (94.1% male) veterans admitted for treatment at one of two VA PTSD Residential Rehabilitation Programs.	Clinical interview and unreported assessment measures	Affirmative response to: “Have you attempted suicide in your lifetime?” (lifetime)	171 (36.23%) reported lifetime SA.^*^	Not reported.	
Legarreta et al. (2015)	95 veterans (77.9% male) who reported at least one lifetime *DSM−5* Criterion A traumatic event.	Checklist created from SCID-IV, Trauma Symptom Index, HAM-D, Profile of Mood States, and methodical query	C-SSRS (lifetime)	24 (36.92%) with PTSD reported lifetime SA.^*^	Not reported	Exclusion criteria of major sensorimotor handicaps, IQ <80, and psychosis. Unvalidated measure of PTSD.
Maguen et al. (2012)	259 male Vietnam veterans in a national survey.	SCID for DSM-III-R	Affirmative response to: “Have you ever attempted suicide?” (lifetime)	Not reported	PTSD was associated with SA in multivariate models that also included demographic variables, depression, SUD, combat experiences, and killing experiences (OR = 5.52, 95% CI = 1.21–25.29, *p* <0.05).	Weighted results.
Monteith et al. (2016)	354 (87.6% male) veterans accessing VHA care.	VA medical record	BSS (lifetime)	Not reported.	There was a significant bivariate relationship between PTSD and lifetime SA, *r* = 0.16, *p* <0.01.	
Ribeiro et al. (2012)	311 military personnel (82.0% male) referred for suicide-focused treatment.	DIS	Clinical interview assessing whether a SA occurred since baseline during one-month follow-up	Not reported.	Accounting for baseline hopelessness, depression, anxiety, drug abuse, alcohol abuse, and insomnia symptoms, PTSD significantly predicted SA, Exp(B) = 6.71; Wald coefficient = 5.83, *p* <0.05.	Results did not account for sociodemographic variables. Sample endorsing SA was small (*n* = 10).
Smith et al. (2015)	832 veterans (gender breakdown not reported) entering a VHA or non-profit PTSD residential program.	Prior primary diagnosis of PTSD (measure not reported)	Interview question: “Have you attempted suicide in your lifetime?” (Yes/No; lifetime)	85 (31.14%) reported a lifetime history of SA.^*^	Did not analyze relationship.	Article reported a total sample of 832; however, suicide-related data were only reported for 273 veterans. Exclusion criteria of active psychosis, unwillingness to discontinue substance misuse, and medical conditions that would hinder/prevent engagement in treatment.

* Calculated using data reported in manuscript.

*BSS, Beck Scale for Suicide Ideation; C-SSRS, Columbia-Suicide Severity Rating Scale; CAPS, Clinician Administered PTSD Scale; DIS, Diagnostic Interview Schedule; DSM-IV, Diagnostic and Statistical Manual of Mental Disorders-IV; FY, fiscal year; HAM-D, Hamilton Rating Scale for Depression; IQ, intelligence quotient; MH, mental health; PHQ-2: Patient Health Questionnaire-2; PHQ-9: Patient Health Questionnaire-9; PTA, posttraumatic amnesia; PSS-I, Posttraumatic Symptom Interview; PTSD, posttraumatic stress disorder; RCT, randomized clinical trial; RR, relative risk; SCID for DSM-III-R, Structured Clinical Interview for DSM-III-R; SCID-IV, Structured Clinical Interview for DSM-IV; SUD, substance use disorder; TBI, traumatic brain injury; VA, Department of Veterans Affairs; VHA, Veterans Health Administration*.

#### KQ1: Prevalence of Suicide Among Military Personnel and Veterans With PTSD

Eight studies were included that reported on the prevalence of suicide among military personnel and veterans diagnosed with PTSD (Table 4). Strength of evidence appeared strong for these studies, as the majority of included studies were determined to have a low overall risk of bias (*n* = 5; Ilgen et al., 2010b; Brenner et al., 2011b; Bohnert et al., 2013; Louzon et al., 2016; Shen et al., 2016). Four studies reported the percent of military personnel or veterans with PTSD who died by suicide, with a range of <0.01–0.30% (Brenner et al., 2011b; Bohnert et al., 2013; Conner et al., 2014; Louzon et al., 2016). An additional four studies reported rates per person-years, ranging from 55.1 to 159.9 per 100,000 (Ilgen et al., 2010b; Black et al., 2011; Bachynski et al., 2012; Louzon et al., 2016), with rates ranging based on timeframe (e.g., per-person year, per-person quarter).

**Table 4:** Suicide among Military Personnel and Veterans with PTSD (*n* = 16).

**Study**	**Population/sample**	**Measurement of PTSD**	**Measurement of Suicide**	**Prevalence of Suicide among those with PTSD**	**Relationship of PTSD to suicide**	**Notes**
Alexander et al. (2014)	150 military service members (94.0% male).	DoDSER	DoDSER	Not reported.	PTSD was not associated with suicide at a bivariate level (OR = 3.34, 95% CI: 0.46–146.53, *p* = 0.229).	The control condition was relatively small (*n* = 27) due to unsuccessful oversampling.
Bachynski et al. (2012)	255 Army personnel (94.5% male) who died of suicide in 2007–2008.	Army Behavioral Health Integrated Data Environment	Army Behavioral Health Integrated Data Environment	Among those with PTSD, the suicide mortality rate per 100,000 person-years was 159.5.	Among those with PTSD, the RR was 5.6 (95% CI: 3.6–8.7, *p* <0.05).	Data were consolidated from the Defense Casualty Information Processing System, the DoD Manpower Data Center, and the Defense Medical Surveillance System. Suicide decedents were compared against the Army as a whole using the Defense Medical Epidemiology Database. The sample of females who died by suicide was small (14; 5.5%).
Black et al. (2011)	874 Army soldiers (94.6% male) who died by suicide from 2001 to 2009.	DMSS	ABHIDE	Among those with PTSD, the mortality rate per 100,000 was 159.9.	The RR for suicide among those with PTSD was 12.61 (95% CI: 9.68 = 16.43, *p* <0.05).	Those who died by suicide were compared to Army personnel over the same time period. Army-wide comparison data were obtained from DMSS and the Armed Forced Medical Examiner System of the Armed Forces Institute of Pathology. The sample of females who died by suicide was small (47; 5.4%).
Bohnert et al. (2013)	3,291,891 veterans (90.0% male) who used VHA care in FY 1999.	VA National Patient Care Database	NDI	226 (.11%) of those with PTSD died by suicide determined to be an “intentional overdose.” ^*^	Unadjusted analyses found PTSD was associated with a relative risk of 4.46 (95% CI: 3.85–5.18, *p* <0.05) as it relates to suicides determined to be an “intentional overdose.”	Veterans were followed through FY 2006. Study classified suicide by overdose as “intentional”, “indeterminate intent”, and “unintentional.” Only “intentional was reported based on purpose of paper.
Brenner et al. (2011b)	7,850,472 veterans (89.8% male) using VHA care from FY 2001 to 2006.	VA medical record	NDI	849 (.30%) of those with PTSD died by suicide between FY 2001-2006.^*^	PTSD diagnosis was associated with higher rates of suicide (*p* <0.0001).	
Conner et al. (2013)	2,962,810 male veterans who used VHA care in FY 1999 and were alive at start of FY 2000.	VA medical record	NDI	Not reported.	After adjusting for age, PTSD diagnosis was associated with increased risk for suicide (HR=1.61; 95% CI:1.34–1.93, *p* <0.05). Risk was elevated in the presence of psychiatric comorbidity (HR = 2.60; 95% CI:2.39–2.82, *p* <0.05).	
Conner et al. (2014)	5,913,648 veterans (90.5% male) using VHA care in FY 2007–2008.	VA National Patient Care Database	NDI	423 (.08%) of those with PTSD died by suicide.	In an unadjusted model, PTSD was significantly associated with suicide (OR = 1.34, 95% CI: 1.21–1.48, *p* <0.001). After adjusting for age, sex, marital status, urban-rural residence, region of country, service era, deployments, new to VHA, depression, alcohol use disorder, drug use disorder, anxiety disorder, bipolar disorder, and schizophrenia, PTSD was associated with decreased risk for suicide (OR = 0.774, 95% CI: 0.69–0.86, *p* <0.001).	
Dobscha et al. (2014)	783 male veterans using VHA care.	VA medical record	State death certificate data	Not reported.	Bivariate associations between PTSD and suicide were not significant, *p* = 0.70.	In this case-control study, all veterans had contact with a VA primary care clinician. Data from all veterans who died in 2009 in an 11-state catchment area were used. Suicide decedents had care from their clinician 6 months prior to death. Controls were matched to cases 2:1 based on same clinician, sex, and age. Females were excluded from analyses due to males comprising 97% of the sample.
Hyman et al. (2012)	2,064,183, in 2005, and 1,981,810, in 2007, active duty military personnel (ranged from 80.3 to 93.9% male).	Military medical record	Reported as “DoD standardized suicide data”	Not reported.	Bivariate OR between PTSD and suicide exceeded 1 for Army (2005: 1.86; 2007: 4.51), and Air Force (2005: 7.40; 2007: 10.57).	The current study was a case-controlled analysis. The relationship between PTSD and suicide was not analyzed for Navy and Marines due to inadequate sampling. Results did not report 95% CI or p-values.
Ilgen et al. (2010a)	5,082 veterans (96.9% male) with SUDs using VHA care in FY 2002–2006.	VA medical record	NDI	Not reported.	Accounting for age, gender, race, and region, PTSD diagnosis was associated with increased risk of violent suicide (OR = 1.33; 95% CI: 1.10–1.62, *p* <0.001) and nonviolent suicide (OR = 2.23; 95% CI: 1.70–2.92, *p* <0.001).	Study examined violent (i.e., firearm, drowning, jumping, use of sharp object, or potentially violent measure) and non-violent (i.e., poisoning) suicide.
Ilgen et al. (2010b)	3,291,891 veterans (96.6% male) who used VHA care in FY 1999 and alive in FY 2000.	VA National Patient Care Database	NDI	Those with PTSD had a suicides rate of 68.6 per 100,000 person-years.	The unadjusted association between PTSD and suicide was significant (HR = 1.93, 95% CI: 1.79–2.08, *p* > 0.05). PTSD remained a significant predictor adjusted for age for both males (HR = 1.84, 95% CI: 1.70–1.98, *p* <0.05) and females (HR = 3.50, 95% CI: 2.51–4.86, *p* <0.05).	Veterans were followed up until death or at the end of FY 2006.
Ilgen et al. (2012)	5,772,282 veterans (90.5% male) receiving VHA care in FY 2007–2008 and still alive at the start of FY 2008.	VA National Patient Care Database	NDI	Not reported.	Accounting for demographics and adjusting at the facility level, proportional hazards regression models found a significant association between PTSD and suicide (HR = 0.36, SE = 0.08, *p* <0.001).	
Kessler et al. (2015)	53,769 military personnel (gender breakdown not reported) with a psychiatric hospitalization.	HADS	Suicide in 12 months after discharge from inpatient hospitalization	Not reported.	PTSD diagnosed during hospitalization was associated with lower odds of suicide in the subsequent 12 months (OR = 0.4; 95% CI: 0.2–0.7, *p* <0.05).	Hospitalizations occurred from 2004 to 2009.
Louzon et al. (2016)	391,492 veterans (92.7% male) using VHA care who received a unique PHQ-9 assessment in FY 2010.	VA medical record	NDI	43 (<0.01%) of those with PTSD died by suicide in FY 2010.^*^ Suicide mortality per 100,000 person-years was calculated as 55.1 based on lifetime PTS diagnosis.	PTSD was not associated with suicide within this sample, *p* = 0.746. Rather, diagnoses of serious mental illness, anxiety, SUD, depression, and “other” diagnoses as well as age, gender, depression severity and SI as measured by the Patient Health Questionnaire-9, and encounter type during Patient Health Questionnaire-9 (i.e., primary care-mental health integration, standard primary care, inpatient mental health, and non-primary care-mental health integration outpatient mental health) were significant correlates of suicide.	
Shen et al. (2016)	3,795,823 military personnel (gender breakdown not reported).	TRICARE medical records	NDI	Suicide rate of those diagnosed with PTSD during the current quarter was 10.26 suicides per 100,000 person-quarter years in comparison to 15.90 and 10.35 per 100,000 person-quarter years for those diagnosed in the previous three quarters or four or more quarters ago respectively. The rate for those without PTSD was 3.94 per 100,000 person-quarter years.	Accounting for sex, race, age, marital status, dependent quantity, rank, Armed Forces Qualifying Test percentile, and military occupational specialty, all mental health diagnoses except PTSD were associated with increased risk for suicide. Specifically, those with PTSD had a lower risk for suicide (hazard ratio = 0.70, 95% CI: 0.57–0.86, *p* = 0.001).	Data were compiled for all U.S. military personnel between 2001 and 2011.
Skopp et al. (2012)	8,782 active duty military personnel (93.6% male).	Military medical record	DoD Medical Mortality Registry	Not reported.	In multivariate analyses, mood disorders, partner relationship problems, and family circumstance problems, but not PTSD (OR: 1.1, 95% CI: 0.75–1.75, *p* > 0.05) were associated with suicide.	This case-control study examined a surveillance period from 2001 to 2009. Personnel were randomly selected and matched 4:1 by service, gender, race, age, entry into service, and years of service.

* Calculated using data reported in manuscript.

*ABHIDE, Army Behavioral Health Integrated Data Environment; CY, calendar year; DMSS, Defense Medical Surveillance System database; DoD, Department of Defense; DoDSER, DoD Suicide Event Report; FY, fiscal year; HADS, Historical Administrative Data System; NDI, National Death Index; PHQ-9: OR, odds ratio; Patient Health Questionnaire-9; PTSD, posttraumatic stress disorder; RR, relative risk; SUD, substance use disorder; VA, Department of Veterans Affairs; VHA, Veterans Health Administration*.

#### KQ2: Association of PTSD With SI Among Military Personnel and Veterans

Nineteen studies reported on the association between PTSD and SI. The majority of included studies were determined to have a high overall risk of bias (*n* = 13), four had a moderate risk of bias (Magruder et al., 2012; Denneson et al., 2014; Monteith et al., 2016; Kimbrel et al., 2018a), and only two studies had a low overall risk of bias (Corson et al., 2013; Finley et al., 2015). Bivariate associations between PTSD and SI were generally significant (significant in ten studies, non-significant in three studies; Table 2). In contrast, results regarding the association between PTSD and SI at the multivariate level were mixed. Of the fourteen studies reporting multivariate results regarding the association between PTSD and SI, seven reported significant associations, and seven reported non-significant associations. All of the studies reporting significant bivariate or multivariate associations between PTSD and SI reported that these were positively, rather than inversely, associated.

#### KQ2: Association of PTSD With SA Among Military Personnel and Veterans

Nine studies examined the relationship between PTSD and SA. Only three had a low overall risk of bias (Brenner et al., 2011a; Gradus et al., 2014; Finley et al., 2015), all of which reported significant associations between PTSD and SA (Table 3). In fact, all but two studies reported significant positive associations at the bivariate and/or multivariate level. In addition, all but one study focused on veterans (versus military personnel).

#### KQ2: Association of PTSD With Suicide Among Military Personnel and Veterans

Sixteen studies examined the relationship between PTSD and suicide (Table 4), with the majority having a low overall risk of bias (*n* = 11; Ilgen et al., 2010a,b, 2012; Brenner et al., 2011b; Skopp et al., 2012; Bohnert et al., 2013; Conner et al., 2013; Dobscha et al., 2014; Kessler et al., 2015; Louzon et al., 2016; Shen et al., 2016). Among these 16 studies, findings were mixed. Nine studies, four of which used multivariate analyses, reported that those diagnosed with PTSD were at greater risk for suicide. Interestingly, in seven studies, five of which used multivariate analyses, PTSD was not a significant predictor of increased risk for suicide (Skopp et al., 2012; Alexander et al., 2014; Dobscha et al., 2014; Louzon et al., 2016), with three of these studies reporting that suicide risk was significantly lower among those diagnosed with PTSD (Conner et al., 2014; Kessler et al., 2015; Shen et al., 2016). When restricting to only studies with low overall risk of bias, results were still mixed, with six reporting significant positive associations (Ilgen et al., 2010a,b, 2012; Brenner et al., 2011b; Bohnert et al., 2013; Conner et al., 2013), three reporting non-significant associations (Skopp et al., 2012; Dobscha et al., 2014; Louzon et al., 2016), and two reporting significant inverse associations (Kessler et al., 2015; Shen et al., 2016).

## Discussion

Given the continued rise in rates of suicide among U.S. military personnel and veterans (Reimann and Mazuchowski, 2018; Department of Veterans Affairs, 2019), understanding the extent to which common mental health diagnoses, such as PTSD, are associated with SI, SA, and suicide is important. Building upon the systematic review by Pompili et al. (2013), the current systematic review provides an update of literature spanning 2010–2018, focused on U.S. service members and veterans. Importantly, this review is inclusive of cohorts spanning multiple service eras, including those who served in the recent conflicts based in Afghanistan and Iraq, a population with notably high rates of suicide (Department of Veterans Affairs, 2019).

### KQ1

This systematic review is among the first to examine the prevalence of SI, SA, and suicide among U.S. military personnel and veterans with PTSD. Attempts to determine rates of SI, SA, and suicide across studies were largely impacted by differences in study methodology. Studies varied in their methods of assessing PTSD and suicide-related constructs (e.g., use of electronic medical records vs. validated semi-structured interviews), as well as in the temporality of these variables (e.g., lifetime vs. past month). This resulted in highly variable rates, especially among studies examining SI.

The majority of included studies examining KQ1 for SI and SA were also rated as having high bias in study methodology. This is likely driven, in part, by the fact that many of these studies were not designed to examine the prevalence of SI or SA. Rather, a number of studies examined PTSD, SI, or SA as secondary outcomes or covariates. In contrast, studies on suicide tended to have a low overall risk of bias, although suicide rates were still quite variable. Stronger methodology and consistent reporting regarding assessment and timeframe of PTSD, SI, and SA is needed for future research to ensure accurate depiction of reported rates.

### KQ2

Research examining if PTSD is associated with SI and S-SDV was also mixed, suggesting a complex relationship. Only PTSD and SA appeared to have a significant association that was largely maintained in the presence of covariates. In contrast, for studies reporting on the association between PTSD and SI or suicide, multivariate findings diverged, with some studies reporting non-significant associations. Further, in some studies, PTSD was even associated with decreased risk for suicide, further complicating conceptualization of this relationship.

Mixed findings regarding the association between PTSD with SI or suicide were maintained even when restricting studies to those with low overall risk of bias. This suggests that while study methodology and rigor are pertinent to synthesizing the literature base, factors outside of study quality may produce varied findings regarding the relationship between PTSD with both SI and suicide.

Several potential explanations can be posited as to why the relationship between PTSD and SI or suicide may be less robust. First and foremost, inclusion of other important correlates of SI and suicide may be more explanatory of risk than PTSD. Several included studies found other variables to be significant correlates of SI, SA, and suicide, including psychiatric comorbidities, psychosocial functioning (e.g., socioeconomic status, social support), and sociodemographic factors. In particular, across several studies, depression was a robust predictor of SI and S-SDV and thus may be more explanatory of suicide risk among U.S. military personnel and veterans with PTSD.

It is also important to note that a number of the studies which reported non-significant relationships between PTSD and SI or S-SDV relied on electronic medical records. There is sizable variability in the diagnostic validity of data obtained from medical records compared to diagnostic interviews (Holowka et al., 2014), suggesting that this may be an important factor to consider when interpreting these findings. The recency (e.g., past-month vs. lifetime) of PTSD, SI, and SA also can be difficult to ascertain from electronic medical records, which may further impact findings.

While our systematic review focused on the presence or absence of a PTSD diagnosis, a dichotomous focus on the presence or absence of a PTSD diagnosis likely does not provide sufficiently nuanced information regarding factors inherent to, or associated with, PTSD that may be driving risk within this population. The clinical presentation of PTSD can vary largely between patients based on heterogenous symptom profiles. In addition, specific symptoms of PTSD, such as guilt, social isolation, and trauma-related beliefs, have been posited as risk factors for suicide among military personnel and veterans (Bryan et al., 2013, 2017; DeBeer et al., 2014; Legarreta et al., 2015; McLean et al., 2017; Holliday et al., 2018a; Borges et al., 2020), but were not the focus of the current review. More research is needed to understand if specific components of PTSD are more strongly related to SI and suicide among military personnel and veterans.

Despite these potential explanations, none provide insight as to why the overwhelming majority of studies on SA found that PTSD was associated with SA, while many studies on SI and suicide did not. Research noting inherent differences in what motivates progression from SI to S-SDV (Nock et al., 2008) may explain some of these differences in findings regarding SI and SA, but would not necessarily explain inconsistencies between findings on SA vs. suicide. One alternate explanation is that, for KQ2, the number of SA studies, particularly low risk of bias studies, were far fewer; as such, inclusion of additional studies may regress toward a more confident understanding of the relationship between PTSD diagnosis and SA. Overall risk of bias for studies on SA were largely rated as moderate to high, leading to potentially spurious findings in comparison to the majority of studies on suicide, which were generally rated as low. This further reinforces the need for additional investigation of the relationship between PTSD and SA, using consistent, sound methodology.

Additional factors underlying S-SDV may also explain variance. For instance, differing means of attempting suicide (e.g., firearms vs. overdose) vary in lethality, as the overwhelming majority of individuals who use a firearm to attempt suicide die (Spicer and Miller, 2000). It is possible that those who survive a SA using a less lethal means differ in their precipitants and drivers of suicide risk in comparison to those who use more lethal means (e.g., firearms). Given that military personnel and veterans have high rates of access to highly lethal means (e.g., firearms; Cleveland et al., 2017) as well as the potential interrelationship between trauma and firearm access (Monteith et al., 2020; Sadler et al., 2020; Simonetti et al., 2020; Stanley et al., 2020), longitudinal research must be prioritized to further understand if PTSD is associated with using different types of lethal means to enact S-SDV.

### Limitations and Future Research

While this systematic review provides important insight into research published since 2010, several factors limit comprehensive inferences regarding this body of literature. A number of studies did not report rates of SI, SA, or suicide specific to those diagnosed with PTSD, precluding inclusion for KQ1. Included study samples were also overwhelmingly male and veteran, which generally reflects the compositions of the U.S. military and veteran populations. Nonetheless, as most studies reviewed did not adequately sample women or report rates or associations separately based on gender, future studies should consider oversampling women to facilitate exploring whether associations between PTSD with SI, SA, and suicide differ by gender.

Moreover, while our review expanded beyond war-related trauma (Pompili et al., 2013) to also include non-combat-related trauma (e.g., military sexual assault), only a limited number of studies specifically assessed and reported on trauma type or focused on non-combat-related PTSD. Because of this, it was not possible to differentiate rates and associations based on types of trauma. Therefore, given research suggesting that risk (e.g., for SI) may differ based on trauma type (Blais and Monteith, 2019), researchers should assess and report type of index trauma among their samples, especially in research pertaining to SI, SA, and suicide.

The current systematic review also focused on PTSD diagnosis, excluding studies relying on self-report symptom inventories (e.g., PTSD Checklist). While these inventories are psychometrically valid and can determine probable diagnoses, they should not be used to infer a formal diagnosis. As this was a departure from prior systematic reviews (e.g., Pompili et al., 2013), it may contribute to differences in findings. A number of studies also used single-item self-report measures of SI and SA. While these items were face valid, their psychometric properties relative to a clinical interview or formal assessment measure is debatable. Further inquiry is needed to understand how method of assessing SI and SA may impact findings.

Included studies were also predominantly cross-sectional. While still informative, such studies were rated as having higher risk of bias as this type of design precludes understanding the temporal relationship between PTSD with SI and SA (i.e., if PTSD precedes subsequent SI and SA). Longitudinal research was limited in this body of literature, and in particular, prospective cohort studies are warranted to further elucidate drivers of suicide risk.

Finally, studies largely differed in the factors that they accounted for in multivariate analyses. Because analytic approaches differed across studies, it is difficult to infer if associations between PTSD with SI, SA, and suicide would have differed based on inclusion of additional variables. As such, researchers should ensure multivariate analyses with PTSD also include correlates of SI, SA, and suicide identified in this review (e.g., depression) to better understand the magnitude and direction of the association with PTSD. Consistent inclusion of these factors would also facilitate future research focused on understanding potential moderators and mediators of the association between PTSD and SI, SA, and suicide (e.g., meta-regression).

## Conclusions

When interpreting findings within the context of these limitations, this systematic review provides insight into the prevalence of SI, SA, and suicide among U.S. military personnel and veterans. Results provide continued, yet tentative, support that PTSD diagnosis is likely associated with SI, SA, and suicide at a bivariate level. Because of this, clinicians should continue to assess for PTSD, as well as associated psychiatric comorbidities (e.g., depression), in the context of suicide risk assessment when working with trauma-exposed U.S. military personnel and veterans. Similarly, clinicians should continue to screen for SI and prior SA when working with military personnel and veterans diagnosed with PTSD.

For many service members and veterans, PTSD may not be the sole driver of suicide risk, as SI, SA, and suicide are likely the result of an accumulation of numerous comorbid risk factors (e.g., depression) and other trauma-related sequelae (e.g., low social support; Lemaire and Graham, 2011). It is important to note that PTSD likely also increases risk for psychiatric comorbidity and decreased psychosocial functioning (Pietrzak et al., 2010; Hefner and Rosenheck, 2019). Therefore, PTSD may increase risk through indirect pathways (e.g., PTSD increases risk for depression, which, in turn, increases risk for suicide; e.g., McKinney et al., 2017).

Given the lack of consistency in multivariate results, further synthesis of the literature remains warranted. Analytic approaches that account for methodological differences between studies (e.g., diagnostic interview vs. electronic medical record) and also synthesize the role of potential covariates and other notable risk factors (e.g., depression) across studies is an integral next step. Additional understanding of specific aspects of PTSD (e.g., guilt, social isolation, trauma-related beliefs) that potentially moderate or mediate the relationship between PTSD diagnosis with SI, SA, and suicide would also further elucidate factors potentially impacting risk among U.S. military personnel and veterans. Identification of specific drivers of risk would be particularly important to inform clinical assessment and evidence-based treatment to prevent SI and S-SDV among U.S. military personnel and veterans with PTSD.

## Data Availability Statement

All datasets generated for this study are included in the article/Supplementary Material.

## Author Contributions

All authors contributed to potential study inclusion, review, and writing of the finalized manuscript.

## Conflict of Interest

The authors declare that the research was conducted in the absence of any commercial or financial relationships that could be construed as a potential conflict of interest.
