# Supplementary material for: Posttraumatic Stress Disorder, Suicidal Ideation, and Suicidal Self-Directed Violence Among U.S. Military Personnel and Veterans: A Systematic Review of the Literature From 2010 to 2018
Source: Front Psychol. 2020 Aug 26;11:1998. doi: 10.3389/fpsyg.2020.01998 (PMC7479813; doi:10.3389/fpsyg.2020.01998)
Supplement: Supplementary file 1 [file Table_1.docx]

Supplementary Table 1

**OVID Medline Search Strategy**

1 exp Self-Injurious Behavior/

2 ((self adj3 (harm or injur* or violen* or destruct*)) or suicid* or selfharm or selfinjur* or parasuicid*).tw,kw.

3 1 or 2

4 exp Stress Disorders, Traumatic/

5 (((posttraumatic or post-traumatic) adj3 stress) or ptsd or ptss).tw,kw.

6 4 or 5

7 exp Veterans/ or exp Military Personnel/ or exp Military Medicine/ or exp Military Nursing/ or exp Hospitals, Military/ or exp "Warfare and Armed Conflicts"/ or exp Naval Medicine/ or exp Military Psychiatry/ or exp Psychology, Military/ or exp Aerospace Medicine/ or exp "United States Department of Veterans Affairs"/ or exp "United States Department of Defense"/ or exp Veterans Health/

8 (veteran* or military or servicemember* or navy or army or "air force" or soldier* or marines or "marine corp" or "marine corps" or corpsmen or corpsman or airmen or airman or "flight crew" or medic or medics or sailor* or submariner* or naval or reserves or infantry* or deployment* or postdeployment* or post-deployment* or war or warfare or warfighter* or combat or "active duty" or "operation enduring freedom" or "operation iraqi freedom" or "operation new dawn" or (deploy* adj1 personnel) or (service adj1 member*) or ((armed or special or defense or defence or security) adj1 force*) or ((national or coast) adj1 guard)).tw,kw.

9 7 or 8

10 3 and 6 and 9

11 limit 10 to english language

12 limit 11 to yr="1980 -Current"

13 limit 12 to "all child (0 to 18 years)"

14 limit 12 to "all adult (19 plus years)"

15 13 not 14

16 12 not 15

17 limit 16 to "animals"

18 limit 16 to "humans"

19 17 not 18

20 16 not 19
